# Activity of Different Types of Cactus Forage on Testicular Function and Morphology of Sheep Subjected to Environmental Heat Stress

**DOI:** 10.3390/vetsci12050492

**Published:** 2025-05-19

**Authors:** Giselle Woolley Cardoso da Silva, Fernanda Carolina Ribeiro Dias, Marli do Carmo Cupertino, Alluanan Adelson do Nascimento Silva, Ângela Maria Vieira Batista, Emanuel Felipe de Oliveira Filho, Francisco Fernando Ramos de Carvalho, Ana Lúcia Figueiredo Porto, Valdemiro Amaro da Silva Júnior

**Affiliations:** 1 Departamento de Medicina Veterinária, Universidade Federal Rural de Pernambuco, Av. Dom Manoel de Medeiros s/n, Dois Irmãos, Recife 52171-900, PE, Brazil; giselle@ufrpe.br (G.W.C.d.S.); alluanan@ufrpe.br (A.A.d.N.S.); ana.porto@ufrpe.br (A.L.F.P.); valdemiro.silvajr@ufrpe.br (V.A.d.S.J.); 2 Departamento de Biologia Estrutural, Universidade federal do Triângulo Mineiro, Rua Vigário Carlos, 100, Bairro Nossa Sra. da Abadia, Uberaba 38025-350, MG, Brazil; 3 Departamento de Biologia Geral, Universidade Federal de Viçosa, Av. P.H. Holfs s/n, Viçosa 36570-900, MG, Brazil; 4 Departamento de Zootecnia, Universidade Federal Rural de Pernambuco, Av. Dom Manoel de Medeiros s/n, Dois Irmãos, Recife 52171-900, PE, Brazil; abatista@ufrpe.br (Â.M.V.B.); franciscarva@ufrpe.br (F.F.R.d.C.); 5 Centro Multidisciplinar de Barra da Universidade Federal do Oeste da Bahia (UFOB), Av. 23 de Agosto, SN, Assunçao, Barra 47100-000, BA, Brazil; emmanuel.fof@ufob.br

**Keywords:** testosterone, thermotolerance, antioxidant activity, reproductive performance

## Abstract

In this study, we aim to analyze whether supplementation with cactus has the potential to minimize the testicular damage caused by heat stress, assess which of the three cactuses would have the best potential, and suggest a possible pathway (oxidative or hormonal) for the action of cactus on the testicular parameter alterations caused by heat stress. Thirty-two male lambs, of the Santa Inês type, not castrated, approximately six months old, and averaging 21.0 ± 2.0 kg body weight, were divided into four groups, as follows: G1, which was fed an elephant grass hay diet (control); G2, with a diet with partial replacement using small cactus forage (*Opuntia cochenillifera*) (SMALL); G3, which was fed Mexican elephant ear (*Opuntia stricta* Haw) (MEE); and G4, which was fed IPA Sertania (*Nopalea cochenillifera* Salm Dyck) (IPA) for 63 days. After slaughter, blood and testicles were removed. The right testicles were fixed for histological analyses, and the left testicles were stored in the freezer for oxidative stress analyses. Serum testosterone, T3, and T4 levels were analyzed. The body weight of animals treated with cactus forage was higher than in the control group. However, the gonadosomatic index did not differ among experimental groups. Heat stress triggered the degradation of testis tissue in all experimental groups. The testicular degeneration process was characterized by tubular atrophy, reduction in germ epithelium height, germ cell vacuolization and necrosis, Sertoli cell vacuolization, germ cell scaling of the tubular fire, and increased intertubular space. The three different cactus forages used in this study had different weaknesses regarding their antioxidant defenses, hormonal levels, and histopathology. However, it is important to highlight that the IPA group had lower qualitative changes in the intertubular areas than the other experimental groups. The testosterone level increased in MEE (*Opuntia stricta*) and IPA groups, while T3 and T4 increased in *SMALL* (*Opuntia cochenillifera*) and IPA groups. The malondialdehyde, an important marker of lipid peroxidation, was reduced only in the IPA group. The testosterone level increased in MEE and IPA groups, while T3 and T4 increased in SMALL and IPA groups. In conclusion, heat stress triggers several histopathologies in testis tissue, and IPA cactus (*Nopalea cochenillifera*) was the most appropriate supplementation for reducing the damages, compared with an elephant grass hay diet or small cactus forage and Mexican elephant ear supplementation.

## Simple Summary

To the best of our knowledge, this is the first study to compare different types of cactus forage supplementation in terms of hormonal and antioxidant enzyme levels, testicular function, and morphology of sheep subjected to environmental heat stress. Heat stress caused the degradation of testis tissue in all experimental groups. The testicular degeneration process was characterized by tubular atrophy, reduction in germ epithelium height, germ cell vacuolization and necrosis, Sertoli cell vacuolization, germ cell scaling, and increased intertubular space. The three different cactus forages used in this study had different weaknesses regarding their antioxidant defenses, hormonal levels, and histopathology. However, it is important to highlight that the IPA-Sertânia (*Nopalea cochenillifera*) group had lower qualitative changes in the intertubular areas among the experimental groups. The testosterone level increased in MEE (*Opuntia stricta*) and IPA groups, while T3 and T4 increased in SMALL (*Opuntia cochenillifera*) and IPA groups. The malondialdehyde, an important marker of lipid peroxidation, was reduced only in the IPA group. In conclusion, heat stress triggers several histopathologies in testis tissue, and IPA cactus was the most appropriate supplementation for reducing the damages compared with an elephant grass hay diet or small cactus forage and Mexican elephant ear supplementation.

## 1. Introduction

The thermal environment is a major climatic factor affecting reproductive parameters and animal production. Animals in heat stress do not exhibit optimum performance and increase energy expenditure because extra energy is required to maintain thermoregulation, and production processes may become less effective. Animals exposed to heat stress are not able to dissipate sufficient heat to maintain homeothermy, leading to an increase in respiration, pulse, heart rate, and body temperatures, resulting in a reduction in food intake, reproduction efficiency, as well as changes in mortality and immune system function [1]. Several possible strategies include feeding them food of good quality and quantity [2].

Sheep have significant importance in semi-arid regions. They produce food with high biological value and valuable raw materials like wool, skin, and manure. In addition, they have strong adaptability and hardiness. In semi-arid regions, sheep and goat farming face two significant challenges: a shortage of food with good nutritional value and the heat stresses these animals are subjected to, which can impair their physical and reproductive performance. The impact of heat stress on reproduction is multifactorial, affecting the fertilizing capacity of sperm through several pathways. The most significant is the increase and accumulation of reactive oxygen species (ROS), compromising motility [3]. Therefore, nutritional management of goat and sheep herds is essential in these production systems [4,5].

Cactus forage was introduced in Brazil to produce the carmine dye, but its potential as forage was later discovered.. Brazil is now the largest producer of cactus forage for animal feed [6]. The Brazilian northeastern region accumulates 66.7% of all sheep farming in the country, and this activity is one of the primary sources of income in the semi-arid region [7,8], of Brazil, with significant export potential. The biggest impasse is the difficulty in increasing herd productivity to reach the necessary number to supply internal and external consumption [9].

In sheep, as in other species, nutrition is directly related to the efficiency of the testicular parenchyma structure and the spermatogenic process. Thus, understanding how different diet types influence these animals’ reproductive tracts is fundamental for economic development [10]. Cactus forage has analgesic, anti-inflammatory, antitumoral, antioxidant, antidepressant, immunomodulatory, neuroprotective, anti-diabetes, and cholesterol-reducing properties [11,12]. Also, this cactaceous influences the reduction in glycemic and lipid levels besides stimulating healing and presenting a positive spermatogenic effect in rats, in some cases reversing sterility [11]. Several studies confirm that using the Opuntia cactus (*Opuntia stricta* Haw) in the nutritional field positively influences the ionic, dietary, and physiological balance [12]. However, almost no research focused on reproductive assessment comparing different types of cactus forage exists [13,14]. The Opuntia cactus type is rich in betacyanins, which stimulate the production of Follicle-Stimulating Hormone (FSH), Luteinizing Hormone (LH), and testosterone hormones and contribute to the maintenance of spermatogenesis [15]. We hypothesize that due to the different components existing in three cactus forage species, they will be able to reduce testicular damage triggered by heat stress, influencing hormone levels, oxidative defenses, and testicular morphology in different intensities or by different pathways. In this sense, this study aimed to evaluate male reproductive system’s function, analyzing possible structural and physiological improvements in testicle tissue from sheep damaged by heat stress conditions by three different types of cactus forage supplementation: MEE (Mexican Elephant Ear (*Opuntia stricta Haw*)), IPA Sertania (*Nopalea cochenillifera* Salm Dyck), and SMALL (*Opuntia cochenillifera*).

## 2. Materials and Methods

### 2.1. Animals and Ethics

Thirty-two 6-month-old mixed breed male Santa Inês crossbred sheep (21.01 ± 2.01 kg), clinically healthy, were housed in individual stalls suspended and slatted at the height of 1.20 m, with an area of 1.5 m^2^, in the goat and sheep sector of the Animal Science Department of the Federal Rural University in Pernambuco state, Brazil. The animals were dewormed and vaccinated and provided with feeders and individual drinking troughs. They were fed twice a day and received water ad libitum. The ethics committee approved on 18 December 2018, the use of animals with License No. 142/2018.

### 2.2. Experimental Groups

Thirty-two 6-month-old mixed breed male Santa Inês crossbred sheep (21.01 ± 2.01 kg) were housed (four groups, n = 8) in individual stalls suspended and slatted at the height of 1.20 m, with an area of 1.5 m^2^, in the goat and sheep sector of the Animal Science Department of the Federal Rural University/Pernambuco state, Brazil. The animals were dewormed, vaccinated, and provided with feeders and individual drinking troughs. The experimental design was completely randomized, using the animals’ initial weight as a covariate. The animals were randomly distributed into experimental treatments. Initially, the data were submitted to normality (Shapiro–Wilk) and homoscedasticity (Levene) tests. Subsequently, analysis of variance (ANOVA) was performed, and the averages were compared by the Student–Newman–Keuls test. The animals were randomly divided into four groups: (i) control standard diet, comprising soybean and corn meals plus elephant grass hay (*Pennisetum purpureum Schum*), and the experimental diets (treatment groups), consisting of the partial replacement of elephant grass hay by: (ii) group 1: small cactus forage (*Opuntia cochenillifera*); (iii) group 2: Mexican elephant ear (*Opuntia stricta Haw*) (MEE); and (iv) group 3: Sertânea (*Nopalea cochenillifera*) (IPA). The ingredients and chemical composition used to produce the diets are described in Table 1. The proportions of ingredients in each experimental diet are described in Table 2.

The forage cactus varieties from the Várzea Alegre farm, located in the municipality of Pesqueira, in the Agreste region of Pernambuco, were harvested after approximately two years of growth and shredded daily in a stationary forage machine, while the elephant grass hay (*Pennisetum purpureum Schum*.) was prepared at the São João do Cariri Experimental Station, belonging to the Federal University of Paraíba (UFPB), and processed in a stationary forage machine, passing through a sieve with an 8 mm screen. Feeding was carried out at 8 am (60%) and 3 pm (40%), and the amounts of food offered and leftovers were measured daily to calculate voluntary consumption, maintaining a level of 15.0% of leftovers. The consumption of dry matter and nutrients was calculated by the difference between the amounts offered and rejected.

The animals, to deworm, received, once, 2 mL of Ivomec^®^ (Ivermectin) for every 20 kg/body weight and a vaccine for clostridial diseases. Environmental conditions that induce heat stress can be calculated using the temperature humidity index (THI), which is determined by ambient temperature and relative humidity (NRC, 1971). During the experimental period, the environmental temperature was within the comfort zone. However, the maximum humidity was higher than 88%, which hinders heat dissipation and increases the animals’ thermal discomfort, according to Eustaquio Filho et al. [16], who considered thermal comfort between 25 and 30 °C and the optimal relative humidity of the air of around 65% for young sheep. Environmental conditions that induce heat stress can be calculated using the temperature humidity index (THI), which is determined by ambient temperature and relative humidity (NRC, 1971). The following formula was used to calculate the temperature humidity index (THI): THI = (0.8 × AT + (RH) × (AT − 14.4) + 46.4), where AT represents the air temperature (°C) and RH is the relative humidity (%) [17]. The temperature humidity index calculated was THI = (0.8 × 28 + (88) × (28 − 14.4) + 46.4, which results in THI = 80.76. According to this index, the animals were under heat stress in this experiment.

After 63 days of treatment, the blood collection was performed by puncture of the jugular vein using a vacutainer. The animals were euthanized with a pneumatic gun, causing brain concussion and exsanguination through the carotid artery. After the death of the animal, the scrotum was sectioned, and the testicles were removed. The associated adipose tissue and epididymis were dissected. The right testicles were removed, weighed, sectioned, and submitted to fixation in a Karnovsky’s solution [18]. The left testicles were stored in the freezer to perform oxidative stress analyses.

### 2.3. Body and Testicular Biometry

The testicles were weighed using a precision scale (BEL Mark 160/0.0001 g). To determine the weight of the testicular parenchyma, the tunica albuginea was removed and weighed separately; this weight was subtracted from the total weight of the whole testicle. Based on body and testicular weights, the gonadosomatic index (GSI) was calculated by dividing the testicular weight by body weight and multiplying it by 100 [19]. The parenchymal somatic index (PSI) was determined by dividing the parenchymal weight by body weight and multiplying by 100 [20]. The parenchymal weight was obtained by subtracting the weight of the tunica albuginea from the total testicular weight (Table 3).

### 2.4. Material Processing for Light Microscopy

The left testes from 8 animals per group were fixed in a Karnovsky’s solution [18] and sent for morphometric and histopathological evaluation. The material was dehydrated in ethanol and embedded in methacrylate resin (Historesin, Leica Microsystems, Nussloch, Germany). Semi-serial sections of 3 μm were made with a rotary microtome (RM 2255, Leica Biosystems, Nussloch, Germany), respecting an interval of at least 39 μm between sections. Three slides with 10 sections each were prepared per animal. In each group, 240 sections were evaluated (3 slides × 10 sections × 8 animals), and in total, 960 sections were evaluated in this study (240 sections per group × 4 groups). The histological sections were stained with toluidine blue/sodium borate (1%). For testicular analyses, digital images were taken with an Axio microscope (Imager.M2m/Zeiss (Oberkochen, Germany)) coupled to a camera (AXIOCam HRc/Zeiss) connected to the ZEN 2 PRO capture software (Blue edition). All images were analyzed using the Image J^®^ program (National Institute of Health, Bethesda, MD, USA).

### 2.5. Morphometric and Histopathological Analysis of the Testicles

Morphological and morphometric analyses were performed under a DM500E Leica microscope with a digital camera attached. The tubular diameter was measured at 100× magnification. Descriptive histopathological analysis was performed according to [21] and degeneration of the testicular parenchyma, atrophy of the seminiferous tubule, desquamation of the germinal epithelium, presence of germinal epithelial cells detached in the lumen, increase in the intertubular space, Sertoli vacuolization, decrease in the size of Leydig cells, and cells in apoptosis or necrosis were described. Only a qualitative analysis of the histopathologies was performed, and from this, categorization was made by an index of lesion intensity. The data are included in the table below the figure showing the histopathologies.

### 2.6. Oxidative Stress

For the oxidative stress analysis, the testicular tissue samples were homogenized in potassium phosphate buffer containing 1 M EDTA (pH 7.4, 0.2 M) and centrifuged (13,800 g at 4 °C for 10 min). We analyzed the superoxide dismutase (SOD), catalase (CAT), glutathione S-transferase (GST), hydrogen peroxide (H_2_O_2_), and malondialdehyde (MDA). All enzyme activities were determined using a spectrophotometer (UV-Mini 1240, Shimadzu, Kyoto, Japan) or an ELISA reader (Thermo Scientific, Waltham, MA, USA).

The activity of superoxide dismutase (SOD) was measured through the protocol of Siddiqui et al. [22]. The catalase activity (CAT) was determined by the rate of a 60-s drop in absorbance of hydrogen peroxide (H_2_O_2_). The production of H_2_O_2_ was measured according to the reaction of Fenton [23] and the activity of the enzyme glutathione S-transferase (GST) through the formation of the glutathione 2.4-dinitrobenzene conjugate (CDNB) [24]. The levels of malondialdehyde (MDA), which is the result of lipid peroxidation, were determined using a TBARS solution (trichloroacetic acid 15%/thiobarbituric acid 0.375%/hydrochloric acid 0.25 M). The total MDA levels in each sample were determined by employing a standard curve from known concentrations of 1,1,3,3-tetramethoxypropane (TMPO) [25]. The samples’ total protein concentration was measured using bovine serum albumin as the standard curve [26].

### 2.7. Hormonal Dosage

Serum samples were collected and stored at −20 °C. At the time of the analysis, they were centrifuged and evaluated according to the electrochemiluminescence method [27] to measure serum testosterone levels. The dosage of Triiodothyronine (T3) and Thyroxine (T4) serum levels was performed through the electrochromic bioluminescence method [28], using the dosage kit according to the manufacturer (Beckham Coulter Access2).

### 2.8. Statistical Analysis

The experimental design was completely randomized, using the animals’ initial weight as a covariate. The animals were randomly distributed into experimental treatments. Initially, the data were submitted to normality (Shapiro–Wilk) and homoscedasticity (Levene) tests. Subsequently, analysis of variance (ANOVA) was performed, and the averages were compared by the Student–Newman–Keuls test. The STATISTICA for WINDOWS 3.11 software was used, with a significant level of *p* ≤ 0.05. All results are expressed as mean ± standard deviation.

## 3. Results

### 3.1. Body and Testicular Biometry

Table 3 presents biometric data. Body weight increased in all three experimental cactus forage groups. Gonadal weight increased only in the MEE and SMALL groups. The gonadosomatic index did not change between groups.

### 3.2. Morphometric and Histopathological Analyses

**Figure 1:**
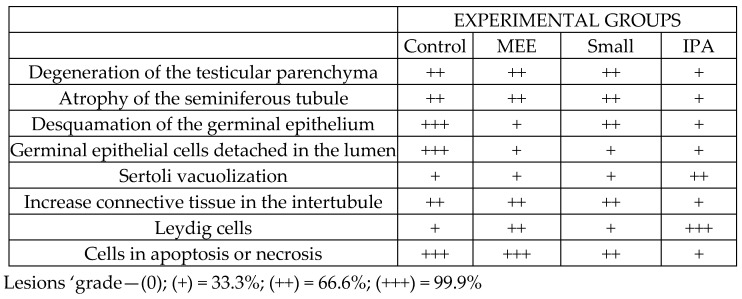
Testicular degeneration process characterized by tubular atrophy, reduction in germ epithelium height, germ cell vacuolization and necrosis, Sertoli cell vacuolization, germ cell scaling of the tubular fire, and increased intertubular space.

According to the morphometric data in Figure 1 the MEE and SMALL groups presented a larger tubular diameter than the other groups, and the IPA group presented a reduction in diameter compared to the other groups. Histopathological analyses found different levels of testicular degeneration in all experimental groups. Descriptive histopathological analysis was performed according to [22], and degeneration of the testicular parenchyma, atrophy of the seminiferous tubule, desquamation of the germinal epithelium, presence of germinal epithelial cells detached in the lumen, increase in the intertubular space, Sertoli vacuolization, decrease in the size of Leydig cells, and cells in apoptosis or necrosis were observed.

However, it is important to highlight that the IPA group had lower qualitative changes in the intertubular areas than the other experimental groups, with evident Leydig cells and no evident connective tissue.

**Figure 2:**
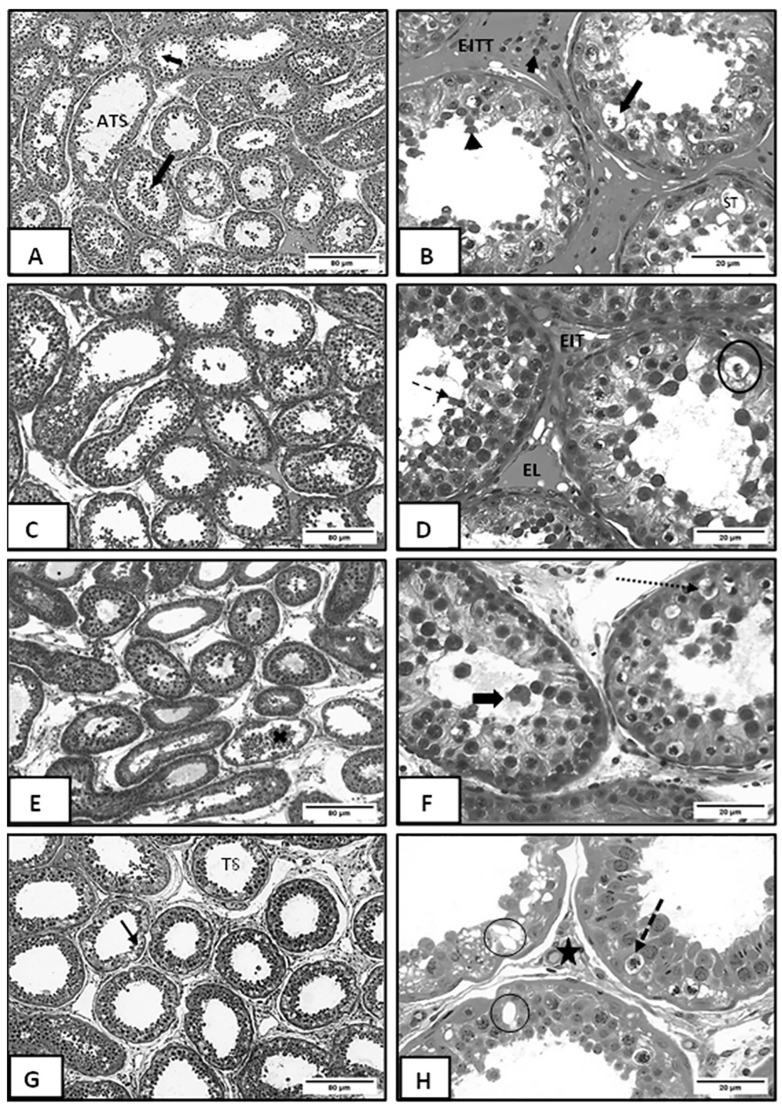
Testicular parenchyma of sheep treated with elephant grass hay and different varieties of *forage cactus*, and categorization by an index of lesion intensity. (**A**) Testicular parenchyma of adult sheep treated with elephant grass hay. Note the seminiferous tubules with reduced germinal epithelium (short arrow and seminiferous tubule atrophy) and shed germinal cells in the tubular lumen (long arrow) (80 µm Bar). (**B**) Higher detail image. Observe germinal cells undergoing cell death (long arrow), shedding of epithelial cells into the lumen (arrowhead), Leydig cell reduction (short arrow), and vacuolization of Sertoli cells (ST). Intertubular space (EIT) (20 µm Bar). (**C**) Seminiferous tubules of adult sheep fed with Ipa Sertânia forage cactus. Note the degeneration of testicular parenchyma and seminiferous tubule atrophy (ATS). Reduction of germinal epithelium (arrow) and shedding of rounded cells into the lumen (star) (80 µm Bar). (**D**) Seminiferous tubules of adult sheep fed with Ipa Sertânia forage cacus. Note the shedding of rounded spermatids from the germinal epithelium (dotted arrow) and germinal cell necrosis (circle). Lymphatic space (EL) and intertubular space (EIT) without evident connective tissue (20 µm Bar). (**E**) Seminiferous tubules of adult sheep fed with small-sized forage cactus. Note the degeneration of germinal epithelium (X) (80 µm Bar). (**F**) Seminiferous tubules of adult sheep fed with small-sized forage cactus. Note the rounded spermatids shed into the tubule lumen (thick arrow) and necrotic germinal cells (dashed arrow). Intertubular space with evident connective tissue (20 µm Bar). (**G**) Seminiferous tubules of adult sheep fed with elephant ear forage cactus. Seminiferous tubules (TS) show degeneration of germinal epithelium (arrow) (80 µm Bar). (**H**) Seminiferous tubules of adult sheep fed with elephant ear forage cactus. Narrowing of intertubular space with evident connective tissue (star), vacuolization of Sertoli cells in germinal epithelium (Circle), and necrosis of germinal cells (dashed arrow) are observed (20 µm Bar).

Categorization by an index of lesion intensity was performed (Figure 1), and photomicrographs of testicular parenchyma in sheep submitted to different types of diet were analyzed (Figure 2).

Figure 2A shows degeneration of testicular parenchyma, atrophy of the seminiferous tubule, scaling of the germinal epithelium, and the presence of germinal epithelium cells detached in the lumen in greater detail. Figure 2B shows an increase in the intertubular space, Sertoli’s vacuolization, a decrease in the size of Leydig cells, and cells in apoptosis, besides showing rounded scaling spermatozoa. Figure 2C shows testicular parenchyma degeneration, atrophy of the seminiferous tubule, reduction of the germinal epithelium, and rounded cells scaling into the lumen. Figure 2D shows the scaling of the rounded spermatozoa of the germinal epithelium and germ cell necrosis. Figure 2E shows the degeneration of germinal epithelium, while Figure 2F shows the scaling of rounded spermatids in the lumen of the tubule and germinal cells in necrosis. Figure 2G shows seminiferous tubules with degeneration of germinal epithelium. Figure 2H shows the narrowing of the intertubular space, Sertoli cell vacuolization in the germinal epithelium, and necrosis.

### 3.3. Oxidative Stress

**Figure 3:**
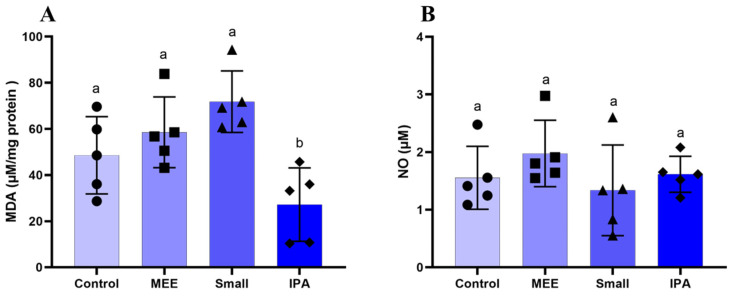
Markers of oxidative/nitrosative stress (**A**) MDA—Malondialdehyde, and (**B**) NO—Nitric Oxide in the testes of SRD sheep fed for 63 days with hay made from elephant grass (Control), Mexican elephant ear cactus (MEE), small cactus, and Sertânia IPA cactus. Data are reported as mean + standard deviation of the mean (n = 6). Different letters between treatments differ significantly at *p* < 0.05 (Student Newman Keuls test).

**Figure 4:**
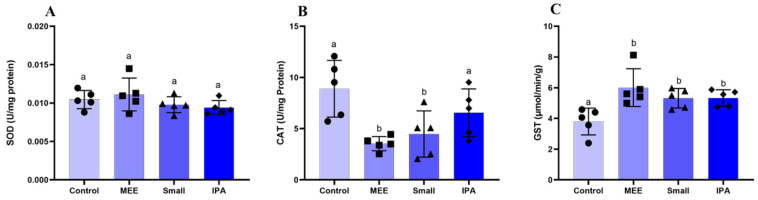
Antioxidant enzyme activity (**A**) SOD—Superoxide Dismutase, (**B**) CAT—Catalase, and (**C**) GST—Glutathione S-transferase in the testes of SRD sheep fed for 63 days with hay made from elephant grass (Control), Mexican elephant ear cactus (MEE), small cactus, and Sertânia IPA cactus. Data are reported as mean + standard deviation of the mean (n = 6). Different letters between treatments differ significantly at *p* < 0.05 (Student Newman Keuls test).

The levels of oxidative stress markers are shown in Figure 3. The MDA levels were reduced only in GI. Nitric Oxide levels did not change. Regarding the activity of antioxidant enzymes, there were no changes in SOD levels (Figure 4A). Catalase activity was reduced in the MEE and SMALL groups (Figure 4B). Finally, glutathione activity was increased in all experimental groups compared to the control group (Figure 4C).

### 3.4. Hormonal Dosage

**Figure 5:**
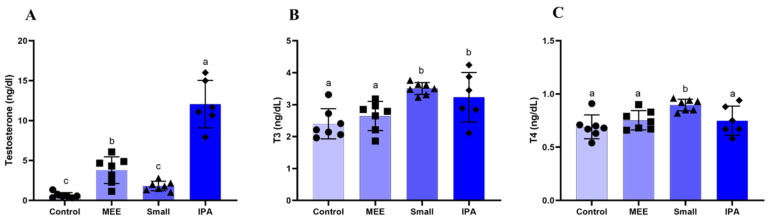
Serum levels (ng/dL) of (**A**) testosterone, (**B**) triiodothyronine (T3), and (**C**) thyroxine (T4) of SRD sheep fed for 63 days with hay made from elephant grass (Control), Mexican elephant ear cactus (MEE), small cactus (Small), and Sertânia IPA cactus. Data are reported as mean + standard deviation of the mean (n = 6). Different letters between treatments differ significantly at *p* < 0.05 (Student Newman Keuls test).

Testosterone levels increased in the MEE and IPA groups compared to the other groups (Figure 5A). T3 levels were increased in the SMALL and IPA groups (Figure 5B), and T4 levels increased only in the SMALL group (Figure 5C).

## 4. Discussion

To the best of our knowledge, this is the first study to compare different types of cactus forage supplementation in terms of hormonal and antioxidant enzyme levels, testicular function, and morphology of sheep subjected to environmental heat stress. Nutrition and temperature are limiting factors in sheep’s gonad development since undernourished and heat-stressed males show late puberty and decreased seminal quality. Testicular weight is an important parameter that defines the animal’s reproductive capacity since it is directly related to sperm production. Thus, testicular weight can be used as a parameter of the prolificacy of male sheep. In the present study, the heat stress triggered different gradations of seminiferous epithelium degeneration. The group treated with elephant grass hay had the most acute lesions, but there were testicular parenchyma damage in all groups.. Animals fed with different cactus varieties gained weight more efficiently than those fed the diet of exclusively elephant grass hay.

The gonadosomatic index (IGS), or percentage of body weight allocated to the gonads, did not differ between groups, indicating a balance between testicular and body weight gain/loss. Testosterone, produced by Leydig cells, in the intertubular area, is essential in maintaining spermatogenesis, sperm lifetime in the epididymis, and accessory sexual glands’ functionality. This hormone also determines the puberty onset and directly influences the appearance of secondary sexual characteristics and libido [29]. In the present experiment, the cactus forage influenced testosterone serum levels in sheep, increasing testosterone levels in cactus supplementation with varietals IPA and followed by MEE. IPA was the group that presented the intertubular area more intact, with less evident connective tissue and more evident Leydig cells.

T3 and T4 hormones increase in animals supplemented with small cactus, and IPA supplementation increases only the T3 hormone. Studies demonstrate these hormones’ direct action on testicular function, stimulating the Leydig cells in the steroidogenic process [30]. Thyroid hormones influence Leydig cells’ maintenance, and therefore, hypothyroidism is linked to decreased plasma testosterone levels [31]. According to the present results, animals fed with cactus forage had higher levels of T3 and T4 when compared to animals fed exclusively with elephant grass hay. Thus, this result corroborates previous studies, since testosterone levels were also found to be higher in animals fed a diet with cactus forage varieties, mainly the IPA varietal.

The most common findings in testicular parenchyma degeneration are atrophy of seminiferous tubules, Sertoli vacuolization, basal membrane thickening, decreased germinal epithelium, and interstitial fibrosis [32]. In the histopathological analysis, different intensities of testicular degeneration were observed in all groups, with the highest intensity observed in the group treated with a diet of exclusively elephant grass hay. The animals treated with cactus forage showed better preservation of the germinal epithelium.

The use of elephant grass hay in both total and partial diets is recurrent. However, there is no correlation between using this type of diet and testicular degeneration or changes in reproductive parameters such as daily sperm production, motility, scrotal circumference, and morphometry [33]. Likewise, up to the present moment, no work related testicular damage to the use of different varieties of cactus forage for sheep

The testicular parenchyma and plasma sperm membrane are rich in polyunsaturated fatty acids related to the fluidity and structure of the sperm membrane, helping the fertilization event. However, these fatty acids are susceptible to ROS (Reactive Oxygen Species) attack, leading to germ cell degeneration and damaging the seminiferous epithelium cycle [34]. The testicular parenchyma evaluations in the experimental groups demonstrated that a normal spermatogenic process was not observed in the injured tubules. The ability to eliminate free radicals by the oxidant potential of cactus forage intermediates of lipid peroxidation was verified due to the increase of antioxidants in the testicles when exposed to injury [35].

Oxidative stress and ROS production are negatively related to testosterone levels in animals [36]. Catalase and glutathione are enzymes that act to prevent free radical formation, both of which are responsible for preventing hydrogen peroxide accumulation [37] Hydrogen peroxide is one of the main ROS, and its presence in cells, even at lower levels, induces apoptosis, causing tissue degeneration [38].

In this experiment, observing a lower level of catalase in animals on a diet of exclusively elephant grass hay was possible. According to Hfaiedh et al. [35], catalase level reduction implies high lipid peroxidation levels. This reaction promotes hydroperoxides, causing the oxidation of fatty acid molecules [39]. The catalase acts by eliminating H_2_O_2_ from the superoxide dismutase reaction, avoiding tissue damage. Thus, when the catalase does not act effectively, hydrogen peroxide accumulation occurs, leading to SOD inactivation [34].

Malondialdehyde (MDA) is the main product of fatty acid oxidation and is a primary marker of lipid peroxidation. The increase in MDA can lead to infertility since, in addition to modifying the membrane structure by interrupting the cellular mechanism, degradation of DNA and protein occurs, leading to a degenerative process [40]. In the present experiment, the diet using IPA cactus significantly reduced testicular lipid peroxidation levels compared to the other groups. The levels of degenerative injuries present in the testicular parenchyma of the group fed with elephant grass hay were more severe than in other groups, since, in addition to demonstrating higher levels of MDA, it had lower catalase production, making its action ineffective in the face of lipid peroxidation. At the same time, the Small promoted increased levels in both groups. Thus, this indicates that the increase in catalase production occurred as a compensatory response to tissue damage suffered due to MDA’s damaging action, indicating a stimulus to the production of enzymes responsible for homeostasis redox.

During the experimental period, the environmental temperature was within the comfort zone. A temperature of 25 °C can be considered the thermal comfort zone for Santa Inês breed sheep in an environment with a relative humidity of 65% [16]. However, the maximum humidity was higher than 88%, which hinders heat dissipation and increases the animals’ thermal discomfort, according to Eustaquio Filho et al. [16], who considered thermal comfort between 25 and 30 °C and the optimal relative humidity of the air around 65% for young sheep. Failure in thermoregulation can lead to heat stress, a condition that compromises sperm quality and increases the risk of infertility in stallions [41]. When prolonged, heat stress also affects dairy cows, as high temperatures impair animal welfare, increase the incidence of diseases, and reduce fertility, which causes serious economic losses to producers [42]. Also, experimental diets, except the one with cactus forage participation, contained 40% concentrate, increasing the internal heat with the caloric increase. Added to the high humidity of the environment, it increased the animals’ thermal discomfort. A similar result was observed by Borges et al. [43], who, working with Santa Inês sheep and three diets (20, 40, and 60% of concentrate), concluded that when the diet passed 20% of concentrate, the physiological parameters required for heat dissipation were impaired, making it difficult to dissipate heat. It became evident that the animals receiving the cactus forage diet improved their thermal comfort by reducing the feeding energy. Also, the cactus presenting high moisture content favors heat loss and electrolytic balance of the animals. Thus, the results found for the animals receiving cactus forage regarding the histopathological, hormonal, morphometric, and oxidative stress variables were variable (Figure 1, Figure 2, Figure 3 and Figure 4 and Table 3).

Testicular degeneration is correlated with thermoregulation control changes [44], which may occur due to changes in environmental temperatures, infections, or in cases of nutritional deficiencies [45]. In the present experiment, all feeding was balanced to ensure that no animal presented any nutritional deficiency, and feeding was not the cause of the injuries, but probably the heat stress. Differences in the animals’ diets, such as urea levels, mineral levels, and the presence of corn, are a possible bias and may impact the parameters evaluated. However, given the above, we can attribute the contributory antioxidant capacity in reproduction to the cactus forage, decreasing the severity of degenerative lesions in all groups and obtaining a positive response in the testicular parameters.

## 5. Conclusions

Cactus forage improved the testicular parenchyma damage triggered by heat stress, possibly due to the reduction of lipid peroxidation. Testicular degeneration was minimized using cactus forage-based diets, being IPA (*Nopalea cochenillifera*) the variety with the highest protective capacity on testicular parenchyma.

## Figures and Tables

**Table 1:** Chemical composition of ingredients.

	Ingredients (g/kg)					
Itens	Elephant C. Hay	Small Palm	IPA-Sertânia Palm	MEE Palm	Soybean Meal	Corn Meal
Dry matter	931.10	118.00	144.30	117.30	899.90	885.50
Organic matter	888.60	890.10	893.60	889.30	913.50	985.70
Mineral matter	111.40	109.90	106.40	110.70	86.50	14.30
Crude protein	57.30	56.90	57.50	59.70	533.80	86.10
Ether extract	20.90	25.90	28.20	30.70	18.10	49.30
Neutral detergent fiber	767.60	289.10	291.90	285.80	232.50	175.20
Neutral detergent fiber	724.60	259.10	252.90	243.20	148.20	156.80
Acid detergent fiber	469.00	142.10	109.60	125.50	24.90	82.00
Hemicellulose	265.60	117.00	143.40	117.80	66.20	131.90
Total carbohydrates	810.50	807.20	807.90	798.90	361.60	850.40
Non-fibrous carbohydrates	75.90	548.20	555.00	555.70	213.40	693.50
Hydrocyanic acid	-	-	0.05762	0.05347	-	-
Total oxalates	0.9671	1.7752	2.0774	2.5706	1.4009	0.9152

**Table 2:** The proportion of ingredients in experimental diets.

	Groups			
Ingredients (g/kg)	Control	IPA	SMALL	MEE
Elephant hay	70.99	25.03	26.31	23.21
IPA-Sertânea Palm	0.00	54.00	0.00	0.00
Palm IPA-Sertania	0.00	0.00	51.65	0.00
Mexican Elephant Ear Palm	0.00	0.00	0.00	57.33
Soybean meal	14.50	19.08	20.05	17.7
Corn	12.85	0.00	0.00	0.00
Urea	0.62	0.56	0.44	0.00
Sheep mineral salt ^1^	0.86	0.95	1.00	0.88
Dicalcium phosphate	0.14	0.33	0.50	0.79
Ammonium sulphate	0.05	0.05	0.05	0.09
Total	100.01	100	100	100

^1^ Manufacturers’ warranty levels: calcium 120 g, phosphorus 87 g, sodium 147 g, sulfur 18 g, copper 0.59 g, cobalt 0.04 g, chromium 0.020 g, iron 1.8 g, iodine 0.08 g, manganese 1.3 g, selenium 0.015 g, zinc 3.8 g, and fluorine maximum 0.87 g. Phosphorus solubility (P) in 2% citric acid is 95% (min.).

**Table 3:** Final sheep bodyweight, testicular weight, and gonad index (IGS) after feeding 63 days with elephant grass hay (Control), Mexican elephant ear (MEE), small palm (SMALL), and Ipa Sertânea (IPA).

	Control	MEE	SMALL	IPA	P
Body weight	24.59 ± 3.20 ^a^	34.89 ± 3.36 ^b^	33.33 ± 1.58 ^b^	34.64 ± 4.20 ^b^	0.001
Gonad weight	0.076 ± 0.045 ^a^	0.19 ± 0.06 ^b^	0.180 ± 0.101 ^b^	0.148 ± 0.079 ^a^	0.04
Gonadosomatic index	0.304 ± 0.183 ^a^	0.53 ± 0.14 ^a^	0.535 ± 0.293 ^a^	0.416 ± 0.176 ^a^	0.1
Tubular Diameter (µm)	189.0 ± 2.6 ^a^	199.9 ± 4.5 ^b^	197.7 ± 3.9 ^b^	176.0 ± 3.8 ^c^	0.04

Values represent means ± standard deviation (significance level: *p* < 0.05) (n = 8). Different letters in the same line indicate the statistical difference.

## Data Availability

The authors will make the data supporting this article’s conclusions available upon reasonable request.
